# An overview of prognostic markers in patients with CLL

**DOI:** 10.3389/fonc.2024.1371057

**Published:** 2024-05-16

**Authors:** Julie Braish, Claudio Cerchione, Alessandra Ferrajoli

**Affiliations:** ^1^ Department of Leukemia, The University of Texas MD Anderson Cancer Center, Houston, TX, United States; ^2^ Hematology Unit, Istituto Romagnolo per lo Studio dei Tumori “Dino Amadori” (IRST), Istituto di Ricovero e Cura a Carattere Scientifico (IRCCS), Meldola, Italy

**Keywords:** chronic lymphocytic leukemia (CLL), next generation sequencing (NGS), prognostic markers, cytogenetics, fluorescence *in situ* hybridization

## Abstract

Chronic lymphocytic leukemia (CLL) is a low-grade B-cell lymphoproliferative disorder. It is the most prevalent type of leukemia in the western countries, with a median age at diagnosis of 70 years. In 2023, it is estimated that there will be 18,740 new cases of CLL, and an estimated 4,490 people will die of this disease. It represents 1.0% of all new cancer cases in the U.S. The rate of new cases was 4.6 per 100,000 men and women per year based on 2016–2020 cases, age-adjusted. Death rates from CLL are higher among older adults, or those 75 and older. The death rate was 1.1 per 100,000 men and women per year based on 2016–2020 deaths, age-adjusted. A common question that patients with CLL ask during their first clinic visit is: “How long will it be before I would need treatment?” Although this might seem like a simple question, the answer is not straight forward. CLL is a heterogenous disease, with a variable clinical course. Some patients may present with an aggressive disease requiring early initiation of treatment, while others have an indolent course and some, having so called smoldering CLL, may never need treatment. The variability in disease course can make predicting disease prognosis a complicated process. This brings forth the importance of establishing prognostic models that can predict disease course, time to treatment, and survival outcomes in such a heterogenous disease. The Rai and Binet staging systems were developed in the late 1970s to early 1980s. They separated patients into different stages based on clinical characteristics and laboratory findings. These simple staging systems are still in use; however, several prognostic markers need to be added for an individualized assessment and, with the recent development of genomic techniques leading to better understanding of CLL at the molecular level, newer prognostic markers have emerged.

## 1 Established prognostic factors

### 1.1 Rai and Binet staging systems

The Rai and Binet clinical staging systems, named after the first authors of the original publications, are inexpensive, easy to use staging systems with good correlation in terms of survival prediction. These two systems were developed in the late 1970s to early 1980s and both divide patients in three major prognostic groups with discrete clinical outcomes (1–5).

The modified Rai staging system, often used in clinical practice nowadays, is a modification of the original Rai classification that reduces the number of prognostic groups from five to three. It defines low-risk disease as patients who have lymphocytosis with leukemia cells in the blood and/or marrow (lymphoid cells >30%) (former Rai stage 0). Patients with lymphocytosis, enlarged nodes in any site, and splenomegaly and/or hepatomegaly are defined as having intermediate risk disease (formerly considered Rai stage I or stage II). High-risk disease includes patients with disease-related anemia (as defined by a hemoglobin [Hb] level less than 11 g/dL) (formerly stage III) or thrombocytopenia (as defined by a platelet count of less than 100 × 109/L) (formerly stage IV) (3, 5).

The Binet staging system is based on the number of involved areas, as defined by the presence of enlarged lymph nodes of greater than 1 cm in diameter or organomegaly, and on whether there is anemia or thrombocytopenia. The areas of involvement considered are (a) head and neck, including the Waldeyer ring, (b) axillae, (c) groins, (d) palpable spleen, (e) palpable liver. The Binet staging system defines stage A as patients having Hb ≥10 g/dL and platelets ≥100 × 109/L and disease involvement in up to two of the above listed areas; stage B as Hb≥10 g/dL and platelets ≥100 × 109/L and organomegaly greater than that defined for stage A (i.e., three or more areas of nodal or organ enlargement); and stage C as Hb of less than 10 g/dL and/or a platelet count of less than 100 × 109/L (2).

With the recent advances in understanding CLL pathophysiology, and the progress in CLL therapy, these two clinical staging systems need to be integrated with other prognostic factors because of the high variability in disease course as some patients with CLL classified as having early-stage disease could rapidly progress if they carry other high-risk features (**Table 1**).

**Table 1:** Prognostic factors in CLL and their clinical relevance.

	CLL Prognostic Factor
**Established Prognostic Factors**	**-Clinical Prognostic Models:** 1- Rai and Benet Staging Systems **-Serum Biomarkers:** 1- Lymphocyte doubling time (LDT) 2- Serum LDH 3- Serum β2 microglobulin , and Tyrosine Kinase **-B-Cell Receptor Biomarkers:** 1- IGHV mutational status 2- B-Cell receptor (BCR) stereotype **-Flow Cytometry Biomarkers:** 1- CD38 and CD49d **-Genetic Aberrations:** 1- FISH mutational panel (Del 17p, Del11q, Trisomy 12, Del 13q) 2- Complex Karyotype 3- Tp53
**Newer Prognostic Factors**	**-Genetic Aberrations:** 1- NOTCH1 2- ATM 3- SF3B1 4- BIRC3
**Prognostic Factors of Potential Interest**	**-Genetic Aberrations:** 1- MYD88 2- Expression of antiapoptotic genes **-Flow Cytometry Biomarkers:** 1- ZAP70 **-Others:** 1- MicroRNA 2- Serum Cytokines 3- Circulating Micro-vesicles 4- Lipoprotein Lipase A and ADAM29 Expression 5- Telomere Length and Telomerase Activity 6- CLLU1 expression

Adapted from “An international prognostic index for patients with chronic lymphocytic leukemia” by The International CLL- IPI working group (6).

### 1.2 Lymphocyte doubling time

Lymphocyte doubling time (LDT) is defined as the period needed for lymphocytes to double in number. The prognostic role of LDT was acknowledged more than 35 years ago by Montserrat et al. and soon after by Molica et al. who investigated LDT in two patient populations and found that a LDT of 12 months or less identified a population of patients with poor prognosis, whereas a LDT longer than 12 months was indicative of good prognosis as substantiated by a long treatment-free period and survival (7, 8) Currently, the iwCLL guidelines include progressive lymphocytosis with an increase of >50% over a two-month observation time or an LDT of less than 6 months as indications to commence treatment (9).

### 1.3 Serum markers (B2 microglobulin, TK)

Two serum markers have been proven for more than two decades to be robust prognostic parameters with independent prognostic value and are still used at the present time.

Beta-2 microglobulin (β2M) is a component of the HLA class I complex on nucleated cells. Patients with serum β2M of < 3.5 mg/L have a substantially longer time to progression, higher rates of complete remission (CR) and longer overall survival (OS) when treated with frontline fludarabine-based chemoimmunotherapy, compared to those with levels of > 3.5 mg/L (10). Thompson et al. reported that in ibrutinib-treated patients at a 6-month landmark unlike FCR treated-patients β2M normalization was seen (11). Additionally, their study showed that β2M normalization was associated with a superior progression-free survival (PFS) in this patient population making it a useful predictor of PFS and clinical decision making (11).

Another serum marker is Thymidine kinase (TK), which is an intracellular protein involved in DNA synthesis. It correlates with disease burden and clinical outcome in patients with CLL. Patients with serum TK values of > 48.5 U/L have been shown to have a more rapid lymphocyte doubling time, a shorter time to progression, and less response to both chemotherapy and targeted therapy (12).

### 1.4 IGHV somatic hypermutation status

The somatic hypermutation status of the rearranged immunoglobulin heavy variable gene (IGHV) remains stable throughout the course of the disease and is an independent prognostic factor that should be determined for all patients with CLL at time of diagnosis. It divides patients into two prognostic and biological sub-groups: IGHV mutated cases (defined as a > 2% difference from the germline nucleotide sequence) with a longer survival from time of diagnosis and longer duration of response when treated with chemoimmunotherapy (more indolent disease behavior) compared to IGHV unmutated cases (defined as 2% or <2% difference CLL w (13).

In 1999, two groups simultaneously reported on the importance of the mutational status of the IGHV. Hamblin et al. sequenced the IGHV genes of the tumor cells of 84 patients with CLL and correlated the mutational status with disease clinical features. They confirmed earlier work by concluding that CLL comprised two separate types of neoplasms arising at different stages of B-cell maturation: a pre-germinal center naïve B cell with unmutated IGHV gene (U-CLL) and a post-germinal center memory B cell with mutated IGHV gene (M-CLL) (13–16). They noticed that patients with U-CLL had a shorter survival than those with M-CLL (13). Damle et al. reported that Ig V gene mutation status and CD38 expression are distinct and reliable prognostic indicators of clinical course and outcome in B-CLL. Their work showed that patients with unmutated Ig V or ≥30% CD38^+^ experienced a worse clinical course than patients in the mutated or <30% CD38^+^ groups (14).

The IGHV mutational status has been very important in the era of chemoimmunotherapy with numerous studies showing longer remissions in patients with M-CLL (17–19) A Multi-institutional study highlighted how patients with M-CLL treated with FCR have a life expectancy that is superimposable to the one of age -match controls and can have extremely prolonged response to therap (18, 20).

IGHV gene mutational status has been incorporated in several prognostic models and clinical tools given its importance in predicting OS and time-to-first-treatment (TTFT) e.g., the CLL international prognostic index (CLL-IPI) (6), the CLL1 prognostic model (21), the International Prognostic Score for Early-stage CLL(IPS-E) (22), and the CLL WithOut Need of Treatment (CLL-WONT) risk score (23). A recent update by the European Research Initiative on CLL (ERIC), highlighted its clinical significance, and discussed in details current recommendations on how to conduct immunogenetic analysis in CLL including how to interpret challenging cases (24).

In patients receiving targeted therapy, the relevance of the IGHV mutational status is still present, albeit somehow less marked. This can be noted in large trials for initial treatment such as RESONATE (25), and ELEVATE-TN (26), where the difference in PFS between patients with M-CLL and U-CLL was noticeable only in the standard chemoimmunotherapy arm and not present in the BTKi-based arm. In patients treated with the combination of venetoclax and obinutuzumab as initial therapy in the CLL-14 study, the IGHV mutational status maintained its importance in both the standard chemoimmunotherapy arm and the BCL2-inhibitor arm with shorter PFS for patients with U-CLL (27). Similarly, 5-year follow up data from the phase 3 MURANO trial showed that patients with relapsed U-CLL treated with venetoclax, and rituximab had higher rates of MRD conversion and disease progression after attaining undetectable MRD at end of treatment (28).

Immunogenetic analysis of the BCR surface immunoglobulins has identified that many patients with CLL share quasi-identical binding sites, so-called “stereotyped” BCRs suggesting the presence of common antigens (29). The antigenic specificity of IgM and IgD is identical, isotype-specific responses are different after IgD and IgM are triggered (30–33).

Several studies have suggested that specific subsets of stereotyped BCRs are associated with distinct clinical features and outcomes, independently of the IGHV mutation status (34).

About 200 different subsets of stereotyped BCRs have been described with subsets 1–8 noted to occur most frequently. Patients with CLL belonging to subset 4 appear to have younger age at diagnosis and experience an indolent course, whereas patients in subsets 1 and 3 experience an aggressive disease course. At the present time there are conflicting reports on whether patients in subset 8 are at a higher risk of developing RS or not (35–39).

Although patients with M-CLL generally have a more indolent clinical course, and display superior responses to certain types of therapy, this is not always the case. In fact, within each somatic hypermutation category, subsets of patients carrying stereotyped BcR exhibit certain biological profiles and clinical outcomes, supporting the notion that BcR stereotypy has the potential to refine prognostication beyond the IGHV gene mutational status (30–33, 40).

Among subsets defined by stereotyped BcR, a specific subset using the IGHV4-39 gene carries a 24-fold increased risk of RS transformation (41). Another example is IGHV3-21, which has correlated with shorter overall survival (OS) regardless of somatic hypermutation status (33).

### 1.5 *TP53* mutations

For several years, *TP53* mutational status was not initially assessed routinely in CLL as it was assumed that *TP53* mutations did not occur in the absence of del (17 p). It later became clear that del (17 p) is usually accompanied by *TP53* mutations in CLL, but they can also occur independently (42).

About 90% of patients with del (17 p) detected by FISH carry a TP53 mutation, and up to 65% of patients with a *TP53* mutation have del (17 p). Patients with a monoallelic *TP53* mutation also have an inferior progression-free and overall survival when compared to patients without this gene mutation. Among patients with TP53 disruption, mutations and deletions occur together in most patients with monoallelic *TP53* mutations accounting for another 40% of patients, whereas monoallelic deletions and mutations with copy neutral loss of heterozygosity account for a minority of patients (43).

The frequency of *TP53* mutations is approximately 10% in treatment-naive patients but increases up to 25%–50% in patients with disease progression and treatment refractory patients indicating that these mutations are mostly acquired and play a role in treatment resistance (43).

Because of its association with dismal outcome, poor response to chemoimmunotherapy, and increasingly important implications on choice of treatment in the era of novel targeted therapy, it is important to establish standardized *TP53* mutational analysis to harmonize testing between centers (44, 45). A recent publication by ERIC, discussed the methodology of NGS and Sanger sequencing for *TP53* analysis, and reviewed the strengths and technical limitations of both techniques to facilitate interpretation of the findings and aid accurate reporting of results (46).

### 1.6 CD38 and CD49d

The expression of CD38 (arbitrarily defined as a cut of ≥ 30% cells expressing CD38) has also been associated with resistance to standard therapy, shorter time to first treatment and shorter OS in patients with CLL (14).

CD49d is an α-integrin subunit (α4) that binds fibronectin and VCAM-1 and plays an important role in nurturing interactions between leukemic B cells and the microenvironment thus influencing growth- and survival- supporting signals and its expression has been found to impact CLL prognosis and represents a potential therapeutic target. The recombinant anti- α4 antibody natalizumab has been studies in the laboratory and shown to reduce migratory ability, interfere with CLL cell recirculation and mobilization of stem and progenitor cells from the bone marrow in vitro, thus having a similar effect to that seen in cells exposed to kinase inhibitors targeting BCR (47).

### 1.7 Zeta activated protein70

ZAP-70 is a molecule involved in T-cell receptor signaling. It is aberrantly expressed in some CLL cases. Several studies have shown a strong association of high ZAP-70 expression with unmutated IGHV genes and BCR function. In all patients in whom at least 20% of the leukemic cells were positive for ZAP-70, IGHV was unmutated, whereas in 21 of 24 patients in whom less than 20% of the leukemic cells were positive for ZAP-70 were found to have mutated IGHV mutations were found (48–50). However, discordance of ZAP-70 expression and IGHV mutational status still occurred in up to 25% of patients and testing for ZAP70 should not substitute performing IGHV mutational status (48–50).

Although used routinely, it is important to note that ZAP-70 is an intracellular antigen with weak expression in CLL, its measurement can be technically challenging and requires internationally defined standards (51). Additionally, other markers e.g., CD38 expression, and IGHV mutational status, have reduced the clinical significance and need for testing for ZAP-70 expression.

### 1.8 Genetic aberrations

Cytogenetic alterations have a key role in CLL prognostication and therapeutic decision making. The low rate of in vitro proliferation rate of CLL cells is a limitation for conventional chromosome banding, whereas fluorescence *in situ* hybridization (FISH) is done in non-dividing cells and has emerged as a relevant, and widely used technique for cytogenetic analysis. Nonetheless, FISH does not provide an overview of the entire karyotype, making both techniques complementary and needed for a full cytogenetic evaluation (9, 52, 53). Both standard cytogenetic and FISH need to be repeated at time of disease progression or initiation of new therapies. Based on genetic alterations detected by FISH, Döhner et al. separated patients into five prognostic subgroups. These subgroups ranging from high to low risk are del(17p) > del(11q) > trisomy 12 > no FISH aberrations > isolated del(13q) (54).

#### 1.8.1 FISH (Del 17p, Del11q, Trisomy 12, Del 13q)

##### 1.8.1.1 Del17(p)

Deletions of the short arm of chromosome 17 (del[17p]) are found in 5% to 8% of treatment-naïve patients. These deletions almost always include band 17p13, where the prominent tumor suppressor gene *TP53* is located (4).

Patients with del17p CLL are known to have an unfavorable prognosis because of shorter time to treatment, shorter remission duration and, once treated, higher rate of disease that can become unresponsive to treatment. As an example, in the CLL8 trial that compared treatment with FCR to treatment with FC, the group with del(17p) had the poorest outcome (progression free survival [PFS] hazard ratio [HR]: 7.49; p<0.0001; overall survival [OS] 9.32; p<0.0001) (4, 55). These patients should never be offered chemo-immunotherapy given that results with target therapies are clearly superior. Targeted therapies have greatly improved the prognosis of these patients; however, the negative impact of del17p remains (55).

##### 1.8.1.2 Del11(q)

Deletions of the long arm of chromosome 11 (del[11q]) frequently encompass band 11q23 harboring the ATM gene, which encodes for the proximal DNA damage response kinase ATM. This abnormality can be found in approximately 25% of chemotherapy-naïve patients with advanced disease stages, and 10% of patients with early-stage disease (10, 54). Patients with del 11q frequently present with bulky lymphadenopathy, and rapid disease progression, and when treated with chemoimmunotherapy experienced a reduced overall survival (54).

##### 1.8.1.3 Trisomy 12

Trisomy 12 is found in 10% to 20% of patients with CLL. Its prognostic relevance remains a matter of debate, but mostly considered intermediate (56). The genes involved in the pathogenesis in trisomy 12 CLL remain unknown. Patients with +12 CLL often have an atypical morphology with a high Matutes score, an increased rate of CD38 positivity and frequently present with an atypical immunophenotype to flow cytometry evaluation (56). Additional reported features in +12 CLL include a higher incidence of thrombocytopenia, tendency to develop Richter Transformation (RT) and a higher rate of second malignant neoplasms (SMN) (4).

##### 1.8.1.4 Normal FISH

Approximately 20% of patients with CLL have a normal FISH which is considered a favorable prognostic finding. Although favorable, outcomes within this group are heterogeneous, bringing forth the value of conventional cytogenetics in this patient population (57).

##### 1.8.1.5 Del13(q)

Deletions on the long arm of chromosome 13, specifically involving band 13q14 (del[13q14]) occur in approximately 55% of all cases making it the most frequently detected abnormality by FISH. Patients whose CLL cells carry this abnormality tend to have an indolent clinical course (54).

#### 1.8.2 Standard karyotyping

The importance of karyotype in CLL was first recognized by Catovsky (58), Juliusson (59), and others, in the 1980s. Recent interest has focused on the relevance of complex karyotype (CK), defined by the presence of at least three chromosome lesions in the same clone, in predicting prognosis (60, 61). Subtypes of CK harboring five or more chromosomal abnormalities (High-CK), and those with major structural abnormalities, also called type- 2 CK are more commonly associated with adverse outcome, shorter time to treatment, and worse response to both chemoimmunotherapy and novel agents (62–64).

Although FISH and standard karyotyping can identify genetic aberrations and prognostic subgroups, these cytogenetic lesions alone do not completely explain disease heterogeneity. Over the last decade, the role of whole genome or exome sequencing (Next-generation sequencing) has greatly expanded, and these analyses have provided a deeper insight into the molecular landscape of CLL.

## 2 Newer prognostic factors

### 2.1 ATM

The ATM gene is located on the long arm of chromosome 11 between positions 22 and 23 (11q22-q23). It codes for a protein that belongs to the superfamily of phosphatidylinositol 3-kinase-realted kinases (PI3K). This protein is activated by DNA double-strand breaks and phosphorylates several proteins that initiate the activation of DNA damage checkpoint signaling pathway, leading to cell cycle arrest, impaired DNA repair or increased apoptosis (18).

ATM gene mutations can be nonsense or missense substitutions, in-frame or frameshift insertions or deletions, and are present in 25% of patients with CLL at diagnosis and are commonly associated with unmutated IGHV, ZAP-70 expression, and presence of del(11q) (30%-40%) by FISH (65).

Austen et al. developed a classification for patients with ATM mutations. They divided them into 4 categories (1): 11q deletions only (2). 11q deletions in association with ATM mutations in the remaining allele (3), heterozygous ATM gene mutation (4), homozygous ATM mutation. They demonstrated that the prognosis of patients with del(11q) and ATM mutations was inferior to patients with isolated del(11q) and these patients had a decreased OS compared with patients without del(11q) (66).

### 2.2 NOTCH1

NOTCH1 is a trans-membrane protein encoded by the NOTCH1 gene located in the chromosome 9q34.3 and involved in the regulation of hematopoietic cell development (18).

NOTCH1 mutations are characterized by frameshift deletions at the C-terminal region (exon 34) leading to a truncated and active isoform of the NOTCH1 protein that accumulates due to defective degradation and constitutive activation of the NOTCH1 pathway (18). It is commonly associated with unmutated IGHV, high ZAP-70 expression, CD38 positivity, and trisomy 12 (67).

It is present in 4%-10% of patients with CLL at the time of diagnosis, 20% of patients with fludarabine-refractory disease, and approximately 30% of patients with Richter’s syndrome (RS) (18).

Patients harboring NOTCH1 mutations usually have a short median time from diagnosis to first treatment and higher risk to develop RS (68).

### 2.3 SF3B1

SF3B1 gene is located on chromosome 2q33.1 and encodes for the subunit 1 (SF3B1 protein) of splicing factor 3b, a nuclear ribonucleoprotein that works in conjunction with other ribonucleoproteins to create the spliceosome responsible for the splicing of messenger RNA (18).

SF3B1 mutations result from missense point mutations, or in-frame deletions, leading to abnormal messenger RNA splicing and enhanced intron retention that alters specific transcripts involved in several cancer-related processes, including cell-cycle control, angiogenesis, and apoptosis (18).

SF3B1 mutations can be found in approximately 10% of patients at diagnosis and 17% of patients with fludarabine-refractory disease (69).

Retrospective analyses have shown that patients with CLL that carry SF3B1 mutations have a shorter PFS and TFS, but not a decrease in OS as compared with patients without this mutation (69).

### 2.4 BIRC3

BIRC3 is encoded in the 11q region, which is deleted in 25% of patients with CLL (54). It is deleted in 83% of patients with del11q CLL. Because almost all del11q CLL are also deleted for ATM, it is unclear whether ATM, BIRC3, or both deletions contribute to the negative prognostic outcome of del11q CLL (66, 70).

Recent work showed that in addition to the loss-of-function mutations, patients with low BIRC3 mRNA expression have a more progressive course of disease due to an altered NF-κB pathway which is associated with increased dependency of CLL cells on the Bcl-2 rheostat, and enhanced sensitivity to Bcl-2 inhibition. Thus, patients with CLL with either low BIRC3 expression or loss-of-function mutations theoretically may benefit from venetoclax-based therapy (71).

## 3 Other prognostic factors of potential interest

### 3.1 MYD88 mutation

The MYD88 gene is located on the short arm of chromosome 3 at position 22 (3p22) and encodes a cytosolic adapter protein that plays a central role in the innate and adaptive immune response. This protein functions as an essential signal transducer in the interleukin-1 and Toll-like receptor (TLR) signaling pathways. These pathways regulate the activation of numerous proinflammatory genes (18).

Trillos et al. noted that patients with CLL and mutated *TLR/MYD88* had a higher frequency of mutated *IGHV* and low expression of CD38 and ZAP-70. Their study also identified a population of young patients (<50 years) with *TLR/MYD88* mutation that showed a favorable outcome and a relative OS comparable to age and gender matched unmutated population (72) (18).

### 3.2 MicroRNA

MicroRNAs (miRNAs) are a group of small noncoding RNAs that play a crucial role in the regulation of gene expression. Alterations in miRNA expression can be found in several types of malignancies, including chronic lymphocytic leukemia (CLL) (73–75). MicroRNA expression has important diagnostic and prognostic value in CLL with some being more commonly identified in patients with low-risk disease, while others being more prevalent in patients with high-risk disease CLL (74).

As an example, MiR-15a and miR-16-1, located at chromosome 13q14, were the first miRNAs identified with prognostic relevance in human cancer. In patients with CLL, they behave as tumor suppressors, target BCL2 and MCL1 and are associated with shorter time from diagnosis to treatment (74).

MiR-34a, targeting ZAP-70 mRNA expression, has been associated with chemotherapy-refractory disease (76). The downregulation of miR-34a and miR-125a upregulation was found to be associated with the development of RS. MiR-155 was reported as the most prevalent oncomiR in B-cell malignancies. It is overexpressed in individual with monoclonal B lymphocytosis and CLL when compared to healthy controls and is a predictor for suboptimal response to therapy and an overall aggressive clinical course in CLL (76–78). Similarly, MiR-29a and miR-29b overexpression leads to aggressive CLL and MiR-129-2 methylation is associated with poor survival in CLL (76).

### 3.3 Expression of Antiapoptotic Genes

The BCL-2 family proteins regulate apoptosis in the mitochondria through the intrinsic apoptotic pathway. These proteins are divided into 3 classes based on sequence homology (BCL-2 homology domains BH1-BH4) and function. The anti-apoptotic proteins that display sequence homology in all BH1 to BH4 domains are BCL-2, BCL-XL, BCL-W, MCL-1, BCL2A1 (BFL-1, A1), and BCL-B. The proapoptotic BCL-2 family members are divided into multidomain effectors (BAX and BAK containing BH1-BH3 domains) and the BH3-only proteins (BIM, PUMA, and NOXA) (79).

Increased expression of the antiapoptotic proteins represents the main factor that accounts for blocking the intrinsic apoptosis pathway. This inhibition of apoptosis is accomplished by sequestering proapoptotic proteins and thus preventing mitochondrial outer membrane permeabilization (79).

Several reports suggest that BCL-2 family proteins could be used as a predictive markers for response to treatment in CLL. The MCL-1/BAX ratio was reported to correlate with resistance to treatment with fludarabine and rituximab. The BCL-2/BAX ratio was also associated with resistance to chlorambucil. BCL-XL levels have also been found to correlate with resistance to various chemotherapeutic agents (80). Resistance mechanisms to targeted therapy with venetoclax have not yet been fully described in CLL patients (63). A small proportion of patients develops mutations in BCL-2. A possible mechanism of resistance in these patients would be upregulation of alternative antiapoptotic BCL-2 family protein members, such as BCL-XL, BCL-W, MCL1, and BCL2A1 and acquired mutations in the BCL-2 BH3 domain and in BAX (63, 81).

### 3.4 Lipoprotein lipase A and ADAM29 expression

LPL plays a central role in lipid metabolism and catalyzes the hydrolysis of chylomicrons and very-low-density lipoproteins. LPL is normally expressed in adipose tissue, cardiac and skeletal muscles, and lactating mammary glands. Low levels of LPL are produced by macrophages, hormone-producing cells in the adrenals and ovaries, certain neuronal cells, thoracic aorta, spleen, testes, lung, and the kidneys (82).

Activation of LPL occurs during differentiation of monocytes into macrophages and involves cytokine regulation. While the expression of LPL in B-CLL seems puzzling and its potential function in leukemic cells is not well understood, it is possible that LPL might play an important role in fatty acid metabolism and energy supply to B-CLL cells. Hence, LPL could be critical for proliferation and survival of the leukemic cells (83).

It has been shown that LPL expression is associated with inferior clinical outcomes and is more commonly found in patients with U-CLL (82).

The ADAM29 gene encodes a member of the disintegrin and metalloproteinase family of transmembrane proteins, which have been shown to mediate cell-to-cell and cell-to-matrix interactions as well as the proteolytic shedding of cell surface molecules (84).

Oppezzo et al. evaluated ADAM29 expression in CLL leukemic cells as compared to normal peripheral B cells and noted absent or very low levels of expression in the latter, suggesting that its over expression could be tumor specific (82). The same group has also reported that ADAM29 is more often expressed in patients with M-CLL and that patients with U-CLL and LPL expression had shorter disease-free survival and overall survival after treatment with chemotherapy compared to patients with M-CLL and/or ADAM29 expression (84).

### 3.5 Telomere length and telomerase activity

Telomeres are nucleoprotein structures that cap the ends of linear eukaryotic chromosomes. Telomere length is a key determinant of telomeric function and short dysfunctional telomeres can drive genomic instability and tumorigenesis in mouse models (85).

There is a link between short telomeres, telomere fusion and adverse outcomes and genomic instability in CLL (86, 87).

### 3.6 CLLU1 expression

CLL Up-regulated gene 1 (CLLU1) is a novel CLL-specific gene located at chromosome 12q22. Its over expression correlates with U-CLL and can predict for shorter time to initiation of therapy and inferior overall survival (88).

### 3.7 Circulating microvesicles

Elevated levels of plasma microvesicles (MVs) have been reported in CLL. MVs are predominantly platelet derived in early-stage patients with CLL but, become B-lymphocyte–derived with disease progression. Higher levels of plasma MV are present in patients with CLL when compared to healthy controls and increased levels of serum MV corelate with disease stage. These findings support a potential us of MV for diagnosis as well as for prognostication (78, 89, 90).

CLL-MVs can stimulate stromal cells to produce more VEGF leading to increased neo-vascularization in the bone marrow and extramedullary tissue. This may modulate the CLL microenvironment in favor of CLL survival and resistance to chemotherapy (91).

### 3.8 Serum cytokines

Several serum cytokines have been studied in CLL. Molica et al. demonstrated an aberrant increase in circulating vascular endothelial growth factor (VEGF) in 17.6% of cases of CLL and correlated increased serum levels of VEGF with risk of disease progression in early stage CLL (92).

T-cell cytokines are also important for the survival and growth of the neoplastic CLL cells. T-cells in patients with CLL produce higher levels of TNF-a and IL-4 than normal T-cells. These cytokines promote proliferation and prevent apoptosis of leukemic B-cells (93). Ferrajoli et al. linked high levels of TNF-a with survival and discussed that elevated TNF-a correlated with advanced Rai and Binet stage disease, higher serum beta2-microglobulin levels, greater CD38 expression, and adverse FISH mutations (94). Serum IL-6 and IL-10 levels are also elevated in CLL, and correlate with adverse disease features (prior treatment, elevated beta (2)-microglobulin, and lactate dehydrogenase, or Rai stage III or IV) and short survival (95).

Sivina et al. evaluated CXCL3 – a chemoattractant that organizes cellular architecture of B-cell follicles and germinal centers- and found that patients with CLL with high serum CXCL3 levels had active and advanced stage disease, and a shorter time to treatment (96).

## Author contributions

JB: Writing – original draft, Writing – review & editing. CC: Writing – review & editing, Data curation, Resources, Supervision. AF: Writing – review & editing, Conceptualization, Writing – original draft.
